# The molecular heterogeneity of sporadic colorectal cancer with different tumor sites in Chinese patients

**DOI:** 10.18632/oncotarget.16176

**Published:** 2017-03-14

**Authors:** Junjie Peng, Dan Huang, Graeme Poston, Xiaoji Ma, Renjie Wang, Weiqi Sheng, Xiaoyan Zhou, Xiaoli Zhu, Sanjun Cai

**Affiliations:** ^1^ Department of Colorectal Surgery, Fudan University Shanghai Cancer Center, Shanghai 200032, China; ^2^ Department of Oncology, Shanghai Medical College, Fudan University, Shanghai 200032, China; ^3^ Department of Pathology, Fudan University Shanghai Cancer Center, Shanghai 200032, China; ^4^ Department of Surgery, Aintree University Hospital, Liverpool L9 7AL, UK

**Keywords:** colorectal cancer, RAS, BRAF, mutation, heterogeneity

## Abstract

**Purpose:**

To assess the biological variability of clinical meaningful molecular markers and their clinical correlations in Chinese patients with colorectal cancer (CRC).

**Materials and methods:**

In this prospective observational study, frequencies and clinico-pathological features of RAS and BRAFV600E mutations, deficiency of DNA mismatch repair (dMMR) were evaluated in patients with colorectal cancer staged I-IV. The molecular heterogeneity between right-sided and left-sided colorectal cancers was studied in our series by classifying patients with different mutations and dMMR status.

**Results:**

Among 400 evaluable patients, mutations in KRAS exon 2, exon 3 or 4, NRAS and BRAFV600E were detected in 36%, 7.5%, 3.5% and 2.5%, respectively. RAS mutations were significantly higher in metastatic CRCs (56.4% vs. 43.1%, p=0.015) and right-sided CRCs (62.5% vs 41.7%, p=0.003). In 212 RAS wild-type patients, V600E mutation was higher in older patients (9.5% vs. 2.2%, p=0.017), women (9.2% vs. 2.2%, p=0.021) and right-sided CRCs (10.5% vs. 3.4%, p=0.06). dMMR was detected in 7.75% of all stages of CRCs, with the highest dMMR rate of 40% in stage II right-sided colon cancer.

**Conclusions:**

By assessing the mutations and clinical correlations of RAS and BRAF genes, and dMMR status, similar RAS mutation, dMMR frequency and lower BRAF mutation was observed in Chinese patients compared to western patients. A distinct molecular heterogeneity was found between patients with right-sided and left-sided CRCs.

## INTRODUCTION

Colorectal Cancer (CRC) is a significant cause of cancer-related morbidity and mortality globally. It is the third most common malignancy in China, and the incidence and mortality rates are continuing to rise in recent years. It has been recognized that colorectal cancer is a biologically heterogeneous disease. The carcinogenesis and development involve distinct pathways, which may result in inactivation of tumor suppressor genes and DNA mismatch repair (MMR) genes or activation of oncogenes [1, 2]. In recent years, with the improved understanding of molecular tumor characteristics, the management of patients with colorectal cancer is changing rapidly. Currently it is possible for clinicians with identified distinct molecular patterns, to make more accurate predictions of prognosis and also treatment response. This progress in understanding molecular changes in colorectal cancer has improved our understanding of the disease and led to more personalized management.

Among the heterogeneous genetic alterations in colorectal cancer, the rat sarcoma viral oncogene (RAS) mutation, V-raf murine sarcoma viral oncogene homolog B1 (BRAF) mutation and DNA mismatch repair deficiency (dMMR) are the main molecular phenotypes studied most extensively and widely applied in clinical circumstances. The molecular features and association of MMR, RAS and BRAF mutations in colon cancers with prognosis have been reported in previous studies. Although the majority of CRCs show chromosomal instability, approximately 15% of cancers develop via an alternative pathway characterized by defective function of the MMR system. These CRCs are known as dMMR tumors, whereas most CRCs have proficient MMR (pMMR). Colon cancers with dMMR have distinct clinical and pathological features, including tumor site, differentiation, treatment response and survival, etc. The Kirsten rat sarcoma viral oncogene (KRAS) mutation status in codon 12 or 13 of exon 2 has been reported in approximately in 40% of patients with metastatic CRC, and recognized as a predictive marker of resistance to EGFR-targeted antibodies in colorectal cancers. Recently, studies have shown that other KRAS (exon 3 or 4) and NRAS mutations are present in another ∼11% of patients, and also associated with resistance to anti-EGFR therapy [3]. BRAF mutation occurs in 5-10% of patients with mCRC with V600E as a hot spot. However, most of reported studies are based on patients in western countries. The possibility that CRC biology differs across races has been suggested by data from population-based studies. To date, data are still lacking in Asian patients with colorectal cancer. Moreover, frequencies of RAS or BRAF mutations were mainly reported in patients with metastatic disease, while dMMR was mainly reported in patients with localized disease. It is important to study the molecular heterogeneity combining all stage patients and compare the difference between Asian and Western patients to establish a more comprehensive molecular profiling of CRC.

In this study, we evaluated the frequencies and clinic-pathological characteristics of RAS mutation, BRAF mutation and DNA mismatch repair expression in Chinese patients with sporadic stage I-IV colorectal cancers, and studied the molecular heterogeneity of patients with different tumor site.

## RESULTS

### Patient characteristics

Of the 400 enrolled patients, 29.5% of patients presented with synchronous metastases, and 31 patients (7.8%) with locally advanced rectal cancer underwent preoperative chemoradiotherapy. The clinicopathological characteristics were shown in Table 1.

**Table 1:** Clinicopathological characteristics of 400 enrolled patients

Clinicopathological characteristics		Number of patients (n=400)	Percentage (%)
Gender	Male	247	61.8
	Female	153	38.2
Age	≥65	131	32.8
	<65	269	67.2
Preoperative CEA	≥5ng/ul	189	47.3
	<5ng/ul	211	52.7
Primary tumor site*	Right-sided*	101	25.3
	Left-sided	299	74.7
Primary tumor site_2	Colon	223	55.7
	Rectum	177	44.3
Histology	Adenocarcinoma	346	86.5
	Mucinous adenocarcinoma	54	13.5
Tumor grade	Low-medium grade	303	75.8
	High grade	97	24.2
p/ypTNM stage**	Stage I	40	10
	Stage II	85	21.3
	Stage III	157	39.2
	Stage IV	118	29.5

*Cancers proximal or distal of the splenic flexure were classified as right-sided or left-sided

**31 patients with rectal cancer underwent preoperative chemoradiotherapy, and ypTNM stage was applied.

### Frequency of MMR status and clinical correlations

We detected dMMR in 31 (7.75%) by IHC analysis for MMR proteins expression. Of the 31 dMMR cancers, 29 cases were found to have either loss of MLH1 or MSH2 protein expression; 3 cases had loss of MSH6 expression, all accompanied by loss of MSH2 expression; 5 cases had loss of PMS2 expression, which were accompanied in 3 cases by loss of MLH1 expression. The prevalence of dMMR status was 12.6% (28 patients) of 223 patients with colon cancer, while only 3 (1.7%) out of 177 patients with rectal cancers were detected dMMR status.

The frequency of dMMR in patients with stage I-IV CRC is listed in Table 2. In colon cancers, there were significantly more patients with dMMR status in stage II colon cancers than other stages of colon cancers. Of 223 patients with colon cancer, the dMMR status was significantly related to mucinous adenocarcinoma (30.8% vs. 8.7% of adenocarcinoma, p=0.0002), high-grade tumor (21.6% vs. 8.6% of low-medium grade, p=0.008) and right-sided colon cancers (19.8% vs. 6.6% of left-sided colon cancers).

**Table 2:** The frequency of dMMR status in stage I-IV patients with different tumor site

Tumor Location	Frequency of dMMR (%)			
	Stage I	Stage II	Stage III	Stage IV
All colorectal cancers	5.0	16.5	7.6	2.5
Right-sided CRC	16.7	40	21.2	5.4
Left-sided CRC	2.9	6.7	4.0	1.2

### RAS/BRAF mutations and clinical correlations

**Figure 1:**
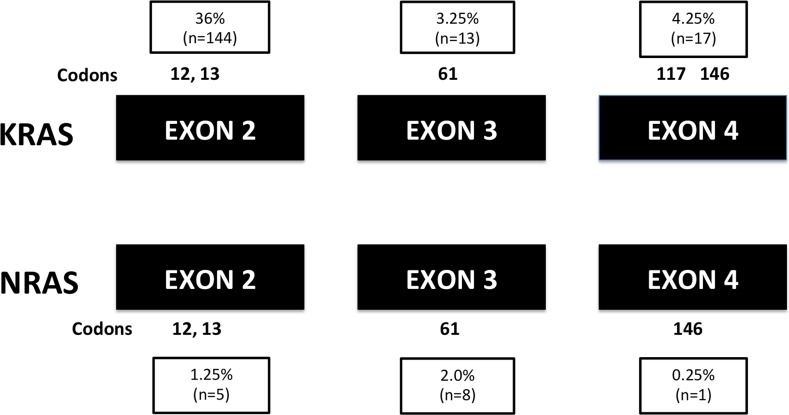
The mutant exons and codons of all RAS mutations (n=400)

Of all 400 patients, 212 patients (53%) had no mutation in KRAS or NRAS gene (RAS wild type). Mutations in KRAS exon 2 (codons 12 or 13) were detected in 144 patients (36%), and mutations in KRAS gene exon 3 and 4 were detected in 30 patients (7.5%); mutations in NRAS gene (exons 2, 3 or 4) were detected in another 14 patients (3.5%). The frequency of all RAS mutation was 47%. The details of mutant exons and codons are shown in Figure 1.

**Figure 2:**
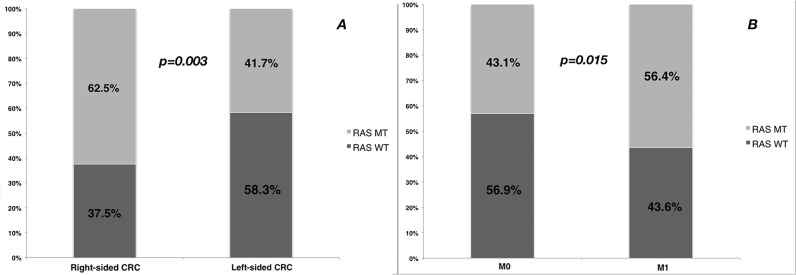
The frequency (%) of RAS mutation by **A**. primary tumor site and **B**. distant metastases.

In stage I-III patients, mutation rates of KRAS exon 2 and extended RAS mutations were 32.7% and 10.3%; in stage IV patients, mutation rates of KRAS exon 2 and extended RAS mutations were 43.7% and 12.6%. In patients with stage I-III disease, RAS mutation was not related to tumor stage, with the mutations rates were 38.5% in stage I, 42.4% in stage II and 44.6% in stage III, respectively. Univariate analysis found that primary tumor site (right-sided vs. left-sided) and distant metastases (M1 vs. M0) were significantly related to RAS mutation status (Table 3 and Figure 2). Multivariate analyses showed right-sided CRC and distant metastases were related to higher prevalence of RAS mutation, with the odds ratios of 2.22 (95% CI 1.39-3.53) and 1.60 (95% CI 1.03-2.49), respectively.

**Table 3:** RAS mutation status by demographic and clinical characteristics in 400 patients with colorectal cancers

Clinicopathological characteristics		RAS WT* (%)	RAS MT (%)	P value
Gender	Male	136 (34)	111 (27.8)	0.294
	Female	76 (19)	77 (19.2)	
Age	≥65	74 (18.5)	57 (14.2)	0.329
	<65	138 (34.5)	131 (32.8)	
Preoperative CEA	≥5ng/ul	94 (23.5)	95 (23.8)	0.216
	<5ng/ul	118 (29.5)	93 (23.2)	
Primary tumor site	Right-sided	38 (9.5)	63 (15.8)	0.0003
	Left-sided	174 (43.5)	125 (31.2)	
Histology	Adenocarcinoma	188 (47.0)	158 (39.5)	0.176
	Mucinous adenocarcinoma	24 (6.0)	30 (7.5)	
Tumor grade	Low-medium grade	158 (39.5)	138 (34.5)	0.798
	High grade	54 (13.5)	50 (12.5)	
MMR status	pMMR	197 (49.2)	172 (43.0)	0.592
	dMMR	15 (3.8)	16 (4.0)	
Distant metastases	M0	160 (40.0)	121 (30.2)	0.015
	M1	52 (13.0)	67 (16.8)	

* Abbreviations: WT, wild type; MT, mutant type; CEA, carcinoembryonic value; MMR, mismatch repair.

BRAF mutation (V600E), was mutually exclusive with RAS mutation, and was only detected in 10 patients, which is 2.5% of all patients and 4.7% of 212 patients with RAS wild type. In 212 patients with RAS wild type, BRAF mutation was found significantly related to older age (9.5% in patients aged≥65y vs. 2.2% in patients aged<65y, p=0.017) and female patients (9.2% in female patients vs. 2.2% in male patients, p=0.021), and the BRAF mutation was higher (approaching significance) in right-sided CRC than left-sided CRC (10.5% vs. 3.4%, p=0.062).

### Molecular heterogeneity of patients with stage I-IV CRCs

**Figure 3:**
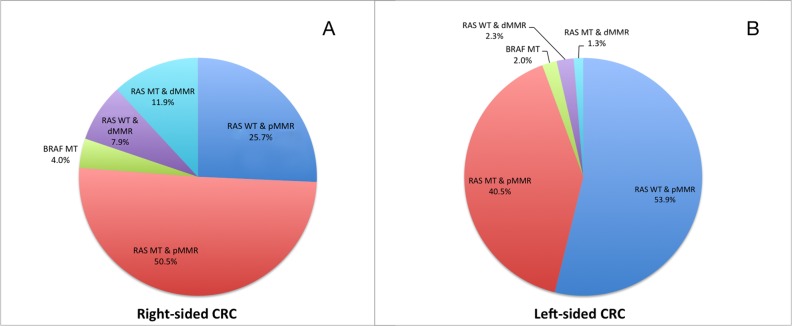
The molecular heterogeneity of patients with stage I-IV CRCs in right-sided (A, n=101) and left-sided (B, n=299) primary tumors

In our series, we classified the patients into five subgroups with different molecular alterations: RAS WT & pMMR (46.8%), RAS MT & pMMR (43.0%), BRAF MT (2.5%), RAS WT & dMMR (3.8%) and RAS MT & dMMR (4.0%). A distinct molecular heterogeneity was observed between patients with right-sided CRC and left-sided CRC (Figure 3).

## DISCUSSION

In the current study, we assessed the mutation of RAS genes and BRAF gene, dMMR and their clinical correlations in 400 Chinese patients with stage I-IV colorectal cancers. A remarkable molecular heterogeneity was also found between patients with right-sided and left-sided CRC.

MMR deficiency is most commonly caused by epigenetic inactivation of the MLH1 gene in sporadic CRCs, and the remainder of dMMR tumors are associated with Lynch syndrome that is caused by germline mutations in MMR genes. Deficiency in DNA repair capacity due to silencing of MMR genes gives rise to the accumulation of abnormalities in short sequences that are repeated up to hundreds of times within the genome (microsatellites). The phenotype is characterized by right-sided location, mucinous cell type and presence of tumor infiltrating lymphocytes. In our study, we also confirmed similar clinicopathological characteristics of colon cancer with dMMR in Chinese patients with sporadic CRCs.

The RAS gene is often mutated in metastatic CRC and the most common of these being KRAS gene mutation. Studies have reported ∼40% of KRAS exon 2 mutation in metastatic CRC, and an additional 10-15% of mutations were detected when extended to all RAS mutation, including KRAS exon 3, 4 and NRAS exon 2, 3, 4 [4–6]. In non-metastatic CRC, the mutation rate of KRAS exon 2 was reported as about 35-37% of patients [7, 8], while the rates of extended RAS mutations were rarely reported. In our series, the frequencies and mutation loci of KRAS exon 2 mutation and extended RAS mutations in Chinese patients were found to be comparable to that seen in western patients, and similar results have also been reported in other Asian patients [9, 10]. In our study, a significantly increased rate of RAS mutations were found in metastatic CRC, compared with non-metastatic CRC (56.3% of RAS mutation in stage IV *vs*. 43% of RAS mutation in stage I-III). Roth *et. al* reported that KRAS exon 2 mutation was related to tumor grade and MSI status in stage II-III colon cancers [7], but RAS mutations were not related to tumor grade or dMMR status in our series.

BRAF V600E mutation Is associated with poor prognosis in patients with mCRC and resistance to conventional chemotherapy, while the prognostic effect of BRAF V600E in non-metastatic CRC remains controversial [7, 11, 12], occurring in about 5-13% of metastatic CRC [7, 13, 14]. However, studies show that racial differences were more significant in BRAF V600E mutation than RAS mutations. Yoon et al. reported that the frequency BRAF mutation was over twice as high in white patients with CRC, compared with Asian patients (13.9% vs. 5.6%) [15]. Teng et al. studied 292 CRC patients from Taiwan after resection of CRC liver metastases, and found 2.1% BRAF V600E mutation [16]. Ye et al. also reported 3.5% of BRAF V600E mutation in 453 patients from China. In our study, the BRAF V600E mutation rate was 2.5%. Otherwise, although low in our series, BRAF mutation was found to be related to older age, female patients and right-sided colon cancer, which was also reported in previous studies [7, 10, 14, 17].

It has long been reported that there are differences in patients' demographic, clinico-pathological features and tumor biology between right-sided and left-sided CRCs [18, 19]. Until recently, analysis of several prospective clinical trials and population-based study proved that these differences could translate into different clinical prognoses [20–22]. In the FIRE-3 study, Heinemann et al. reported significant survival superiority in KRAS wild-type metastatic CRCs with left-sided primary tumors, compared to right-sided primary tumors [22]. The CALGB/SWOG 80405 study reported no significant difference in overall survival and progression-free survival when patients with KRAS wild-type metastatic CRC were treated with bevacizumab or cetuximab, both in combination with leucovorin/fluorouracil/oxaliplatin (FOLFOX) or leucovorin/fluorouracil/irinotecan (FOLFIRI) [23]. However, retrospective analysis of the CALGB/SWOG 80405 study found that the median survival was significantly longer in patients with left-sided primary tumors than that of right-sided primary tumors. Moreover, the sidedness of the primary tumors seemed to have translated over into different treatment responses to targeted therapy [20]. Data from a population-based study also confirmed that right-sided stage III and IV CRCs had significantly inferior prognoses compared to left-sided CRCs [21]. Although these findings haven't changed our current treatment strategies, it indicated that the side of primary tumor might be a surrogate for biological variability across the large bowel, which can also be explained by the different embryonic origins of right-sided colon and left-sided colon and rectum. In our series, by studying several crucial molecular markers in colorectal cancer, we found significant molecular heterogeneities between right-sided and left-sided CRCs. The frequencies of dMMR, RAS mutations and BRAF mutation were all more commonly detected in right-sided CRCs than left-sided CRCs. Furthermore, CRCs patients were divided into five groups with these clinical markers, and a great difference of molecular heterogeneity was clearly observed In the Pie Charts comparing right and left-sided CRCs.

There were limitations of our study. The survival outcomes haven yet to be obtained, and so any correlation between the molecular heterogeneity and patients' survival remains unknown. The limited case numbers is another limitation to the detailed analysis of differences betwee different stages. However, our study further confirms the biological variations of CRCs between different races.

## MATERIALS AND METHODS

### Patient population

Between May 2014 and November 2015, 400 evaluable patients (screened from 431 patients) with sporadic colorectal cancers were prospectively but non-consecutively collected for molecular analysis in the Department of Colorectal Surgery, Fudan University Shanghai Cancer Center. All study patients underwent surgical resection of their primary tumors in our institution. Reasons for excluding 31 patients included hereditary colorectal cancer (15 cases), unavailable tissue for genetic testing (11 cases) and non-adenocarcinoma lesions (5 cases).

This study was reviewed and approved by local Institutional Review Boards. All patients gave informed consent for the use of their cancer tissue blocks for molecular analyses.

### Immunohistochemistry (IHC)

The IHC assay for the MMR proteins expression was performed using the fully automated BenchMark ULTRA platform (Ventana Medical Systems, Inc., Tucson, Arizona, USA) using a comprehensive panel of four primary antibodies, including MLH1 (M1), MSH2 (G219-1129), PMS2 (EPR3947) and CONFIRM MSH6 (44) antibodies (Ventana Medical Systems, Inc., Tucson, Arizona, USA). Nuclear staining of normal tissue next to the tumor or lymphocytes in the stroma served as internal positive controls. In tumors, dMMR was interpreted as follows - loss of an MMR protein (MLH1 or MSH2 or PMS2 or MSH6) expression was defined as the absence of nuclear staining of tumor cells. Each slide was examined by at least two experienced pathologists.

### DNA extraction and mutations screening

Areas of tumor-rich were scraped from 5-μm unstained serial sections after confirmation by hematoxylin and eosin-stained slides. Extraction of genomic DNA was carried out using the QIAamp DNA Mini Kit following the manufacturer's protocol (Qiagen, Valencia, CA, USA). DNA content was quantified using NanoDrop ND-1000 (Nanodrop, Wilmington, DE, USA).

DNA samples were amplified for regions of KRAS exon 2, 3, and 4, NRAS exon 2, 3, and 4, and BRAF exon 15 by polymerase chain reaction (PCR) using the Real-time PCR master mix (TOYOBA, Osaka, Japan). PCR reactions with the primers listed in Supplementary Table S1 were conducted in a 25ul volume containing 5–10ng DNA on the Mastercycler System (Eppendorf, Hamburg, Germany). Circling conditions for KRAS, NRAS and BRAF were (1) 94°C for 7 min, (2) 94°C for 30 s, (3) the relevant annealing temperature for each primer for 30 s (shown in Supplementary Table S1), (4) 35 cycles at 72°C for 30 s, and (5) 72°C for 5 min. The PCR products were analyzed on a 2% Biowest agarose gel (GENE, Hong Kong, China) and purified with a QIAquick PCR Purification Kit before sequencing (Qiagen, Valencia, CA, USA). Bidirectional sequence was performed using ABI 3730XL using a BigDye Terminator v. 3.1 Cycle Sequencing kit (Applied Biosystems, Carlsbad, CA, USA). The positive samples were confirmed by 3 independent experiments.

### Statistics

The clinicopathological correlations of dMMR, RAS mutations and BRAF mutation were analyzed by *chi-square* test in univariate analyses. Multivariate analysis was performed using logistic regression and the odds ratios were recorded. P value <0.05 was considered statistically significant.

## CONCLUSION

Our study assessed the mutation of RAS genes and BRAF gene, dMMR and their clinical correlations in Chinese patients with colorectal cancers. Similar RAS mutation and dMMR frequencies were found in Chinese patients as compared to what has been observed in Western patients, while the frequency of BRAF mutation was lower in Chinese patients. A distinct molecular heterogeneity was found between patients with right-sided and left-sided CRCs. Future studies are required to further understand the mechanism of this biological variability.

## SUPPLEMENTARY MATERIALS TABLES


