# Supplementary material for: The molecular heterogeneity of sporadic colorectal cancer with different tumor sites in Chinese patients
Source: Oncotarget. 2017 Mar 14;8(30):49076–83. doi: 10.18632/oncotarget.16176 (PMC5564750; doi:10.18632/oncotarget.16176)
Supplement: Supplementary file 1 [file oncotarget-08-49076-s001.pdf]

The molecular heterogeneity of sporadic colorectal cancer with different tumor sites in Chinese patients

SUPPLEMENTARY TABLE

Supplementary Table S1: The primers of the PCR reactions of RAS genes and BRAF gene

		Primer sequence	Size (bp)	T_A (°C)
KRAS exon 2	Forward	5′-GGCCTGCTGAAAATGACTG-3′	173	58
	Reverse	5′-GGTGCAGGACCATTCTTTG-3′		
KRAS exon 3	Forward	5′-CTGTGTTTCTCCCTTCTCAGG-3′	281	58
	Reverse	5′-TGCATGGCATTAGCAAAGAC-3′		
KRAS exon 4	Forward	5’-TGACAAAAGTTGTGGACAGGT-3′	247	58
	Reverse	5′-TGTTACTTACCTGTCTTGTCTTTGC-3′		
NRAS exon 2	Forward	5′-CAGGTTCTTGCTGGTGTGAA-3′	144	58
	Reverse	5′-CACTGGGCCTCACCTCTATG-3′		
NRAS exon 3	Forward	5′-CCCCAGGATTCTTACAGAAAA-3′	244	58
	Reverse	5′-CCCCATAAAGATTCAGAACACA-3′		
NRAS exon 4	Forward	5′-AGGGAGCAGATTAAGCGAGT-3′	198	58
	Reverse	5′-CAAACTCTTGCACAAATGCTG-3′		
Braf exon 15	Forward	5orward5n 15CAAATGCTGATGCi-3′	237	59
	Reverse	5everse5n 15CAAATGCTGATGCi		
